# Tannins of Constant Structure in Medicinal and Food Plants—Hydrolyzable Tannins and Polyphenols Related to Tannins

**DOI:** 10.3390/molecules16032191

**Published:** 2011-03-04

**Authors:** Takuo Okuda, Hideyuki Ito

**Affiliations:** 1 Emeritus Professor, Okayama University, Tsushima-naka, Okayama 700-8530, Japan; 2 Division of Pharmaceutical Sciences, Okayama University Graduate School of Medicine, Dentistry and Pharmaceutical Sciences, Tsushima-naka, Okayama 700-8530, Japan; Email: hito@cc.okayama-u.ac.jp

**Keywords:** tannin and polyphenol, type A tannin, type B tannin, classification, biological and pharmacological properties

## Abstract

In addition to the commonly used classification as hydrolyzable tannins and condensed tannins, tannins can also be categorized into two other types: polyphenols of constant chemical structure (Type A) and polyphenols of variable composition (Type B). Both types of tannins and related polyphenols account for a large part of plant polyphenols, but accurate structure-activity correlations on a molecular basis can be determined mainly for type A compounds, among which are hydrolysable tannins such as the ellagitannins and their oxidized congeners, some gallotannins, epigallocatechin gallate, caffetannins, *etc.* Among the activities determined on a molecular basis are the chemical, biological and pharmacological actions such as superoxide anion scavenging, apoptosis, antitumor, anti-EVB, anti-MRSA and anti-plasmin inhibitory activities, *etc.*, in addition to their fundamental activities, *i.e.*, binding to proteins, large molecular compounds and metallic ions, and antioxidant activities. Some structure-specific activities were found for the condensation of dehydroellagitannins with co-existing compounds under mild conditions, and the host-mediated antitumor actions of ellagitannin oligomers. Structures and activities of metabolites of geraniin, a dehydroellagitannin, were revealed. Some stilbenoids and phlorotannins of firm structures have been known to have many activities similar to those of the type A tannins.

## 1. Introduction

Tannins are polyphenols sometimes called plant polyphenols [1], although originally the name tannin was given to the plant extracts exhibiting astringency, without knowing their chemical structures. The features distinguishing tannins from plant polyphenols of other types are basically the properties of the former: binding to proteins, basic compounds, pigments, large-molecular compounds and metallic ions, and also anti-oxidant activities, *etc.* These features of tannins lead to qualitative and quantitative analytical differences between tannins and other polyphenols. Unlike the analysis of polyphenols in general, quantification of tannins is based on their binding activity referred to above. The classical hide powder method is based on the binding with animal skin protein, and the RA (relative astringency) and RMB (relative affinity to methylene blue) determinations [2] are based on the binding with blood and methylene blue respectively, under controlled pH.

These properties of tannins are based on their chemical structures having two or three phenolic hydroxyl groups on a phenyl ring, in a molecule of moderately large size. Tannins were once classified into two groups: pyrogallol type tannins and catechol type (or catechin type) tannins, according to the polyphenol groups in their molecules. Then, the developments in tannin chemistry led to the renaming of these two groups to hydrolyzable tannins and condensed tannins [1,3,4]. Caffetannins, labiataetannins and phlorotannins were also referred to tannins [3,4,5] The isolation of bio-active stilbenoids, among which was a monomer piceatannol is regarded as responsible for the tannin activity of the bark of spruce tree [6], and various resveratrol oligomers, and also phlorotannins from brown algae, exemplified by monomeric eckol [7], expanded the field of tannins and related polyphenols to these groups of compounds in the last few decades. At the same time various biological and pharmacological activities related to the health effects of tannins with a variety of chemical structures, including those of small molecular size, have been found [8,9,10,11]. As for the molecular size, (−)-epigallocatechin gallate (EGCG) and (−)-epicatechin gallate (ECG), the main “tannin” in green tea, are examples exhibiting the properties of tannins in spite of their rather small molecules. They exhibit binding activities towards proteins and other substances and appreciable antioxidant activities [9,10,11], besides their antitumor effects [12,13,14].

On the other hand, in the past there was a vague concept that tannins are intractable mixtures of phenolics of rather large molecules, which is inconsistent with the findings exemplified above. There are many tannins that are rather small molecules having distinct chemical structures, and nevertheless showing the typical properties of tannins.

Constancy in the chemical structure and composition is essential for characterizing the biological and pharmacological properties of any target compound, while these properties of tannins described in the past often lacked confirmation of such constancy of their composition. Recent advances in tannin chemistry starting with isolation of the ellagitannins [4,7,10,15], led to additional categorization of tannins and related polyphenols into two types: type A, with constant structures, and type B of variable composition.

## 2. Type A Tannins and Related Polyphenols of Constant Structures and Compositions

All ellagitannins, exemplified by crystalline monomeric geraniin [16] and dimeric agrimoniin [17] are type A tannins. (−)-Epigallocatechin gallate (EGCG) and (−)-epicatechin gallate (ECG), which are the easily isolable main components of green tea tannin, are exceptional members of the polyhydroxyflavan family exhibiting tannin activity, despite their small, stable structure molecules [2,7]. Resveratrol [18] and piceatannol [19], which are polyhydroxystilbene monomers, are both of interest in food research because of their presence in grape skins and wine, in spite of their low concentration. Eckol [20] and diechol [20], phlorotannins in brown algae, can also be counted along with the above described compounds as the members of type A tannins.

*1,2,3,4,6-Penta-O-galloyl-β-D-glucose*, obtainable by partial hydrolysis of the labile depside linkages of gallotannin mixtures from Chinese gall (gall of *Rhus javanica*), or nutgall (Turkishgall, gall of *Quercus lusitanica*) [21], can be counted as a type A tannin. Hamamelitannin, a digalloylhamamelose first isolated from a *Hamamelis* species [22], although not responsible for the protein-binding activity of the extract from this plant, may also be structurally regarded as a type A tannin.

While most of the polyhydroxyflavan oligomers belong to the type B tannins, which are variable in their structure and composition in plants and extracts, as described in Section 3.2, ellagitannin oligomers can generally be counted as type A tannins because of consistancy of production of each oligomer in a plant species, regardless of season, their ease of their isolation, and the amount of each compound found in a species of plant.

The discovery of these isolable compounds of the type A, particularly those responsible for the tannin activity of each plant species, allows the accurate chemical, biological and pharmacological analysis of these tannins.

### 2.1. Gallotannins of Type A (Figure 1)

Gallotannins have core structures in which a carbohydrate or quinic acid is galloylated at several hydroxyl groups. The galloyl groups in these core structures are often further galloylated *via* depsidic likages forming type B gallotannin mixtures. However, some type A gallotannins with stable structures can be isolated.

*Acertannin*. (2,6-digalloyl-1,5-anhydro-D-glucitol). This crystalline compound, first isolated from the leaves of *Acer ginnala* [23], can be structurally counted among the type A gallotannins, although its binding activity is low [2]. The tri- and tetragalloyl derivatives having depsidically linked galloyl groups [24,25] showed significant binding activity [25].

*Hamamelitannin.* This digalloylhamamelose, first isolated from a *Hamamelis* species [22] is not responsible for the protein-binding activity of the extract from the plant, but structurally it can be regarded as a type A tannin. Several galloyl esters of hamamelose were isolated from *Castanea* species (Fagaceae) and *Sanguisorba* species (Rosaceae) [26,27], and galloyl esters of other sugars and cyclitols were obtained from the plants of various families, including Fagaceae.

**Figure 1:**
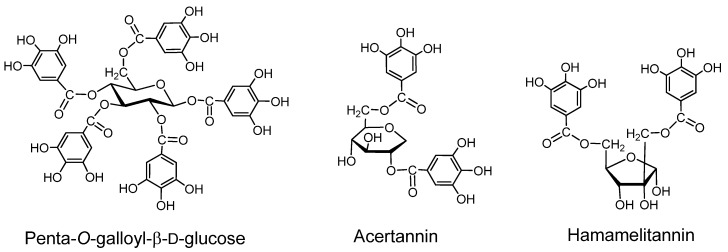
Gallotannins.

### 2.2. Ellagitannin, Dehydroellagitannins and Their Oxidatively Transformed Analogs

Ellagitannins have hexahydroxydiphenoyl (HHDP) group(s), and dehydroellagitannins have a dehydrohexahydroxydiphenoyl (DHHDP) group which is an oxidatively modified congener of the HHDP group esterifying the hydroxyl groups in the carbohydrate or cyclitol core [1,4,7,10]. Some of dehydroellagitannins, such as geraniin, forms crystals [7]. There are also some analogs having a polyphenol group of further oxidized structures, exemplified by chebuloyl group [28]. These tannins generally belong to the type A tannins. The gallotannins and the tannins biogenetically derivable from gallotannins can be classified into four types: I (gallotannin), II (ellagitannin), III (dehydroellagitannin) and IV (oxydatively transformed dehydroellagitannin). The tannins biogenetically producible from these tannins can be classified into four types: I+, II+, III+ and IV+ [29]. These tannins are found in the following subclasses in the Cronquist’s system of plant evolution: Magnolidae (I, II and II+), Hamamelidae (I, II, II+, III and IV), Rosidae (I, I+, II+, III, III+ and IV) and Dilleniidae (I, II, II+, III and IV+) suggesting a correlation between their oxidative transformation and plant evolution starting from Magnolidae [10,29]. However, often all of the tannins of these types are called ellagitannins. They are relatively stable compounds isolable from the plant without suffering destruction of original chemical structures. The oxidative structural transformation of these tannins starting from gallotannins to highly oxidized ellagitannin derivatives [10,30] is obviously correlated with the plant evolution system [29]. More detailed classification is possible for the tannins derivable from gallotannins and ellagitannins including their oligomers with increasing structural diversity [7,10,29].

Among the biological and pharmacological activities of these tannins are host-mediated antitumor activities, and antimicrobial activities exemplified by those against *Helicobacter pylori*, antibiotic-resistant bacteria and *Leishmania donovani* [7,8]. Some examples of ellagitannins in the wide sense and their noticeable chemical reactions are described in Section 2.2.2.

#### 2.2.1. Ellagitannin and dehydroellagitannin monomers and their oxidized congeners

**Figure 2:**
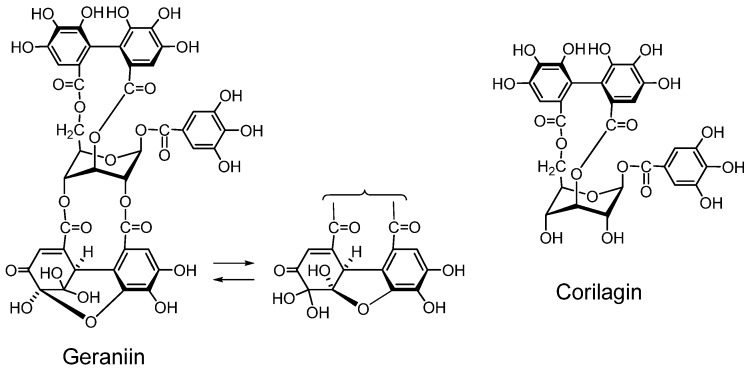
Geraniin and corilagin.

*Geraniin* (Figure 2) A dehydroellagitannin first isolated from *Geranium thunbergii*, which is one of the most frequently used medicinal plants in Japan, is mainly applied to intestinal disorders, and is an official medicine registered in the Japanese Pharmacopoeia. This crystallizable compound accounts for over 10% of dry weight of leaf of the plant. Its structure, with a *R*-HHDP, *R*-DHHDP and a galloyl group, was elucidated by chemical and spectroscopic means [16] and conformed completely to the X-ray chrystallography result [31]. Synthesis of some DHHDP esters was reported [32].

This compound was also found in high content in all examined *Geranium* species, and also in many species of plants of Hippomaneae, Acalypheae and Euphorbiaceae, *etc.* in the order Geraniales [33]. Geraniin is also one of the most notable compounds in the correlation of the tannin structures with plant evolution, because of its location in the plant evolution chart is correlated with the oxidative transformation of hydrolyzable tannins [29].

Unlike commercially available “tannic acid”, the action of geraniin on human tongue and mucous membrane is very mild. Because of the firm and chracteristic chemical structure of geraniin was determined in early stages of its investigation, in addition to the wide application of *Geranium thunbergii* as a folk medicine in Japan, this tannin has often been among a target of biological and pharmacological investigations [34,35,36,37,38].

*Corilagin* (Figure 2). Corilagin is an ellagitannin, forming the primary part of the structures of several ellagitannins and dehydroellagitannins exemplified by geraniin and chebulagic acid. It can be produced by their partial hydrolysis occurring upon extraction of geraniin and chebulagic acid in boiling water, which causes variations in the biological activity of the tannin such as inhibition of of mutagen mutagenicity [34]. A total synthesis of corilagin was reported [39].

**Figure 3:**
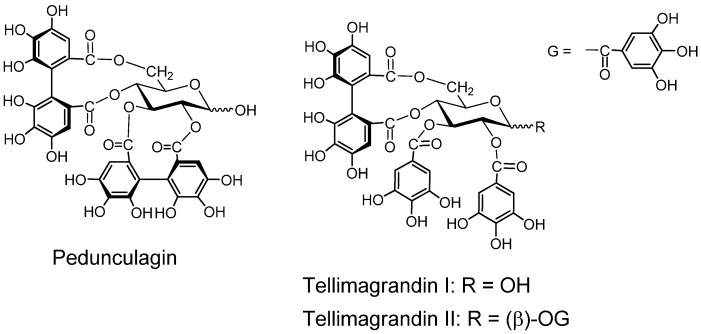
Ellagitannins and oxidized analogs.

*Pedunculagin* (Figure 3). Pedunculagin, found in *Casuarina* and *Stachyurus* species, *etc.* and having exclusively two *S*-HHDP groups on the glucose core [35], is a typical ellagitannin. *Tellimagrandins I and II* (Figure 3). These ellagitannins having an *S*-HHDP group [40], were first isolated from *Tellima grandiflora* [41].

**Figure 4:**
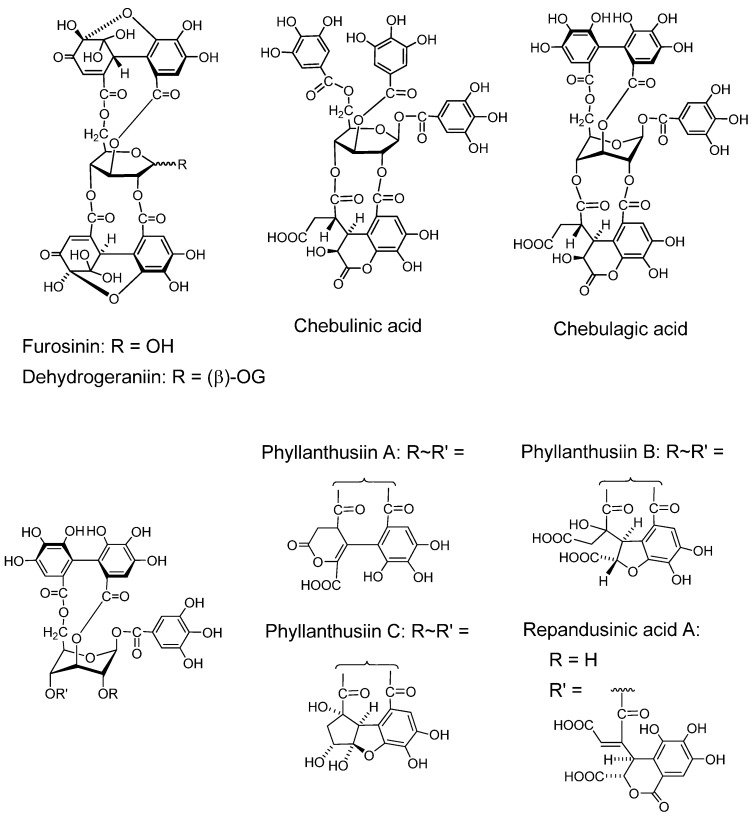
Dehydroellagitannins and oxidized congeners.

*Furosinin* and *dehydrogeraniin* (Figure 4). These dehydroellagitannins have further oxidized structures derived from geraniin, having a *R*-DHHDP group in place of the *R*-HHDP group found in geraniin. While dehydrogeraniin having a galloyl group at *O*-1 of the glucose core exists as a mixure of four isomers, furosinin, lacking the glucose core, is present as a mixture of eight isomers, although these isomeric structures are those of a single compound [42].

*Chebulinic acid* and *chebulagic acid* (Figure 4). These compounds, isolated from myrobalans (fruits of *Terminaria chebula*) [43] have a polyphenolic group which is regarded as a product of further oxidation of the DHHDP group [28,44].

*Phyllanthusiins A, B and C,* and *repandusinic Acid A* (Figure 4). Four different polyphenolic groups producible by oxidation of the DHHDP group were found in these tannins isolated from *Phyllanthus flexuosus* [45].

#### 2.2.2. Condensation of dehydroellagitannin with coexisting compound under mild conditions (Figure 5)

Because of high reactivity of the DHHDP group, dehydroellagitannins easily condense with several types of compound under very mild conditions. Condensations of this sort are considered to occur in the plant cells or in plant homogenates, as found for the occurrence of ascorgeraniin.

*Condensation of Geraniin with Ascorbic Acid (Ascorgeraniin)*. Geraniin condenses with ascorbic acid under moderately aqueous acidic conditions or in aqueous methanol at room temperature to yield ascorgeraniin (elaeocarpusin) [46,47]. Ascorgeraniin produced in the plants was isolated from *Geranium thunbergii* [46] and *Euphorbia watanabei* [48]. It is probable that analogous condensation can occur in any plant producing dehydroellagitannins.

*Condensation with Acetone (Phyllanthusiin D)*. Phyllanthusiin D, a condensate of geraniin with acetone, was isolated from acetone or aqueous acetone homogenate of *Phyllanthus flexuosus* [45] and *P. amarus* [49], and also from suspension cultures of *Geranium thunbergii* [50]. Since this condensate was produced when a solution of geraniin in dry acetone with a small amount of trifluoroacetic acid was refluxed, phyllanthusiin D is regarded as an artifact produced during the extraction, but it is likely that such condensation with acetone can occur upon homogenization of any plant producing dehydroellagitannins.

*Condensation with o-phenylenediamine*. In aqueous acetic acid at room temperature, the DHHDP group in dehydroellagitannins condenses with *o*-phenylenediamine yielding a phenazine derivative after a short time [51]. This condensation was applied to terchebin [52] and geraniin [53], and is considered applicable to the other dehydroellagitannins.

**Figure 5:**
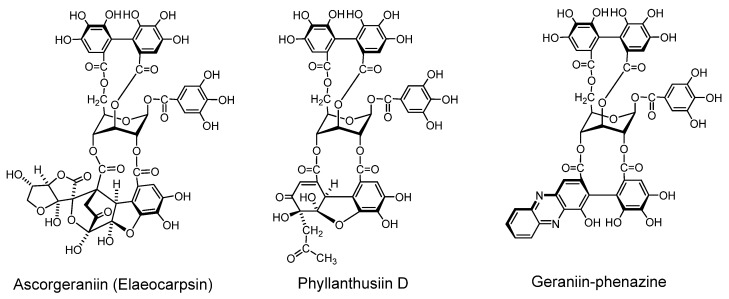
Condensation products from geraniin.

#### 2.2.3. Ellagitannin oligomers with notable pharmacological activities

As found for agrimoniin, the first oligomer of ellagitannin isolated from plants, each one of several oligomers is the main component in a species of plant. They are notable by their pharmacological activities, exemplified by the host-mediated antitumor activity. Up to pentamers of the ellagitannin oligomers have been isolated.

##### 2.2.3.1. Ellagitannin and dehydroellagitannin oligomers (Figure 6)

*Agrimoniin*. This dimer was isolated along with potentillin, a monomer unit of agrimoniin, and pedunculagin from *Agrimonia pilosa* and *Potentilla kleiniana*, both belonging to the Rosaceae [54]. The α-glucosidic linkage in agrimoniin and potentillin is noticeable. Agrimoniin is one of the oligomers exhibiting host-mediated anti-tumor activity [55]. Agrimoniin was also found in other *Agrimonia* species*,* and also in *Rosa*, *Potentilla* and some other Rosaceae genera [56].

*Gemin A*. This dimer was isolated from *Geum japonicum* [57], and is found specifically in *Geum* species, while agrimoniin occurs in several genera of Rosaceae [56]. *Coriariin A*. This dimer having potent host-mediated antitumor activity was isolated from *Coriaria japonica* [58].

**Figure 6:**
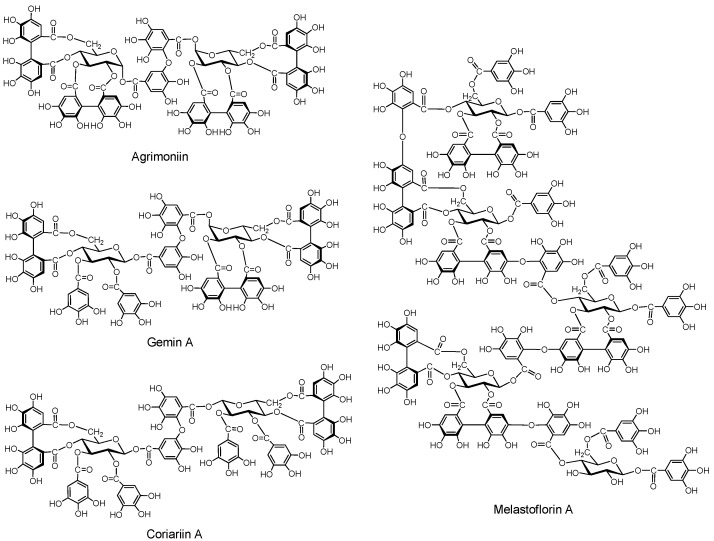
Ellagitannin oligomers.

*Higher Oligomers (Pentamers)* (Figure 6). Pentameric ellagitannins melastoflorins A–D, were isolated from *Monochaetum multiflorum,* a melastomataceous plants [59], although yields of the higher oligomers, trimers, tetramers and pentamers, are generally lower than those of the dimers. The presence of hexamer and heptamer was proposed based on HPLC-MS analysis [60]. The host-mediated anti-tumor activities of the higher oligomers were rather less potent than those of several dimers [55].

##### 2.2.3.2. Macrocyclic Oligomers (Figure 7).

Macrocyclic dimers and trimers with potent host-mediated antitumor activity have been isolated.

*Oenothein B.* This macrocyclic dimer was isolated from *Oenothera erythrosepara* [61], and *Epilobium* species of the Onagraceae, *Lythrum anceps* [62], and *Woodfordia fruticosa* of the Lythraceae, along with *woodfordin C* with an analogous macrocyclic structure [63]. Oenothein B is one of the most active compounds showing host-mediated antitumor activity among the ellagitannin oligomers [64]. *Oenothein A* and *woodfordin D*, macrocyclic trimers of mutually analogous structures with similar antitumor acivities were isolated from *O. biennis* and *W. fruticosa*, respectively [65].

**Figure 7:**
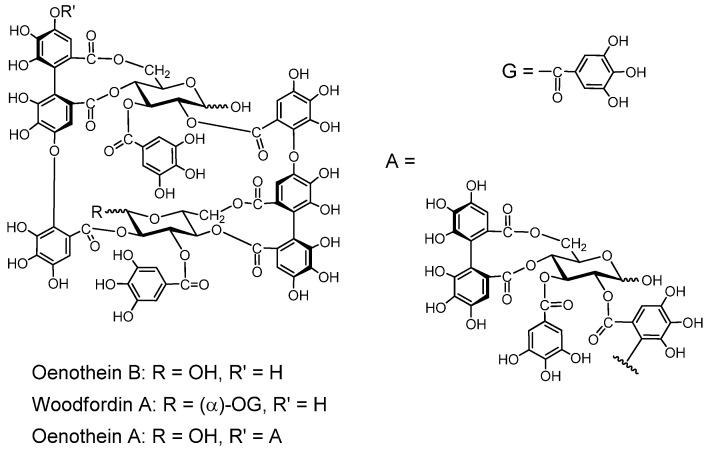
Macrocyclic ellagitannin oligomers.

##### 2.2.3.3. *C*-Glucosidic Ellagitannins and Complex Tannins

The *C*-glucosidic tannins exemplified by casuariin, casuarinin and stachyurin from *Casuarina* species [35] have a polyphenol group linked *via* a carbon-carbon linkage to *C*-1 of an open-chain glucose. The *C*-glucosidic ellagitannins in the heartwood of oak and chestnut trees are mixtures, mainly composed of the monomers vescalagin and castalagin, *etc.* and of the dimers roburins A–D [66]. These tannins change their quantity in wines aging in barrels [67], and their extraction kinetics were investigated [68].

Complex tannins such as the camelliatannins A and B from *Camellia japonica* [69] and malabatrin A from *Melastoma malabathricum* [70] have a flavan at *C*-1 of an open-chain glucose. Complex tannins can further be classified into flavano-ellagitannins having a flavan-3-ol, and flavono-ellagitannins having a flavonoid glucoside, each connected to *C*-1 of glucose in the hydrolyzable tannin moiety through a carbon-carbon linkage.

### 2.3. Transformations of the Type A Tannins in Plants, during Extraction and upon Ingestion of the Medicines and Foods Containing Them

While the type B tannins, particularly condensed tannins, change their structures seasonally, depending on the mode of plant growth and of storage of plant material, each type A tannin compound of constant structure is always obtainable from the specified plant producing it. Precise inversigation of the structural transformations occurring during extraction and application as medicines and foods can be therefore performed for most type A tannins.

#### 2.3.1. Transformation of hydrolyzable tannin structures in young leaf of a woody plant

Type A tannins produced in herbaceous plants basically retain their chemical structures until their leaves decay, but those in some woody plant transform their structures in the early parts of the season. Unlike the structural changes of polyhydroxyflavans, those of the type A tannins occur stepwise along specific biosynthesis route, and can be traced clearly. An example of seasonal transformation of the chemical structures of tannins is that seen in the young leaves of *Liquidambar formosana* starting from gallotannins and ending in ellagitannins [71].

#### 2.3.2. Transformation of ellagitannin structures during extraction

Medicinal plants have traditionally been used by decoction of crude drugs, *i.e.* by extraction of dried plant in boiling water. The type A tannins in the plants, retaining the original structure during drying, often undergo hydrolysis during decoction, as found for the hydrolysis of geraniin yielding corilagin, ellagic acid and brevifolincarboxylic acid [34,72]. These polyphenols produced by hydrolysis often have noticeable health effects exemplified by the anticarcinogenic [73] andantineoplasia [74] effects of ellagic acid.

#### 2.3.3. Transformation of ellagitannin structures upon ingestion

Various pharmacological activities of tannins mentioned in Section 2.5 suggest the importance of their beneficial effects in human health, and the structural transformation of ellagitannins in the wide sense during extraction as referred in Section 2.3.2 implies occurrence of further transformation of their structures upon ingestion, like those observed for most of metabolized compounds. The effect evaluation of ingested tannins in medicines and foods should therefore include that of structurally transformed tannins. The structural transformation of geraniin in rats is taken as an example.

**Figure 8:**
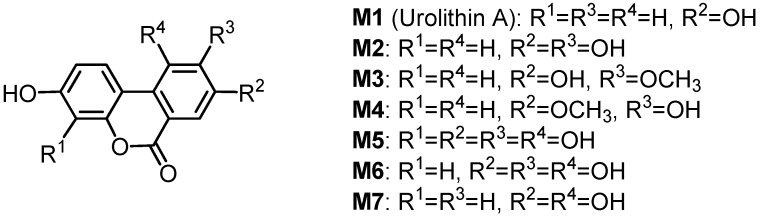
Metabolites of geraniin.

Seven urinary and intestinal microbial metabolites were isolated in rats after the ingestion of geraniin, and their structures were determined to be the dibenzopyran derivatives **M1**–**M7** (Figure 8) [75]. The antioxidant activities of the four major metabolites **M1**–**M4** prepared by chemical synthesis were evaluated by using oxygen radical absorbance capacity (ORAC) methods. These four metabolites exhibited more potent antioxidant activities in the ORAC assay than intact ellagitannins, such as geraniin and corilagin. The ORAC of plasma increased with the increase of the metabolites’ concentration in plasma after the oral administration of geraniin to rats [76]. These findings suggest that the metabolites may generally contribute to the health benefits of ellagitannins in the body as antioxidants. Glucuronide formation of several of these metabolites in the serum and urine of a sheep given *Terminalia oblongata* leaves containing chebulagic acid, punicalagin and teroblongin (1-α-*O*-galloylpunicalagin) was reported [10].

### 2.4. Polyhydroxyflavan Gallates

(−)-Epigallocatechin gallate (EGCG), a monomer accompanied by (−)-epicatechin gallate (ECG) is an exceptional member of the polyhydroxyflavans since they exhibit considerable binding and other tannin-like activities in spite of their small molecules. Their activities are examples of the significant promotion of the biological effects by galloylation [2].

**Figure 9:**
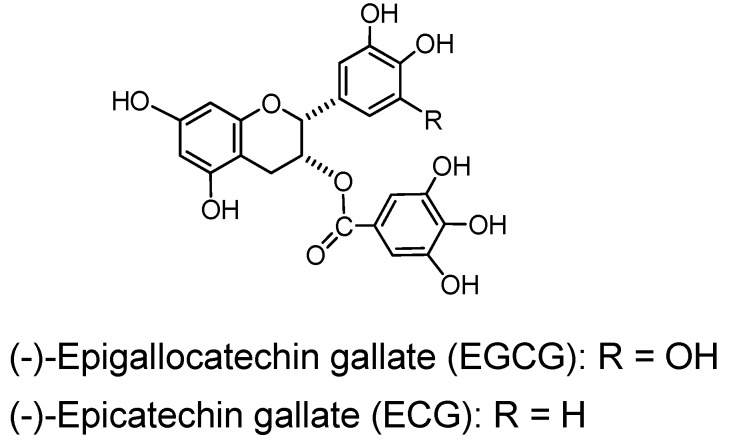
Green tea tannins.

*(−)-Epigallocatechin gallate (EGCG)* and *(−)-epicatechin gallate (ECG)* (Figure 9). (−)-Epigallocatechin gallate (EGCG), accompanied by smaller amounts of (−)-epicatechin gallate (ECG), is the main component in the green tea tannins and largely responsible for the tannin activities of green tea, *i.e.* binding to protein and pigments [2], antioxidant [77] and astringency on the tongue. These activities of EGCG were comparable in potency to average activities of tannins in general. It also exhibited siginificant antitumor activities, and there have been accumulated data of antitumor activities of EGCG [78,79,80]. The activities of ECG, although somewhat lower, were similar to those of EGCG.

### 2.5. Pharmacological Activities of Ellagitannins and Monomeric Polyhydroxyflavan Gallates

The biological and pharmacological activities of tannins determined precisely to date are mainly those of the type A tannins.

#### 2.5.1. Antimicrobial activities

The synergistic effects of ellagitannins with antibiotics against antibiotic-resistant bacteria is one of the most noticeable antimicrobial activities of tannins [81]. Corilagin and tellimagrandin I markedly potentiated the activity of β-lactams against methicillin-resistant *Staphylococcus aureus* (MRSA) [82]. Tellimagrandin I and rugosin B markedly lowered the minimum inhibitory concentrations (MICs) of oxacillin against the MRSA strains [83]. Oenothein B, a macrocyclic ellagitannin dimer also suppressed the antibiotic resistance of methicillin-resistant *Staphylococcus aureus* (MRSA). Potent anti-human immune-deficiency virus (HIV) activities were found for the dimeric ellagitannins oenothein B, coriariin A and agrimoniin [84].

#### 2.5.2. Antitumor activities

Several kinds of anticarcinogenic activities of tannins have been found. The inhibition of tumor promotion by tannins that has been most extensively investigated is that of EGCG [12]. Antitumor activities were also found for various tannins from Asian plants [13].

##### 2.5.2.1. Inhibition of the mutagenicity of carcinogens

Several ellagitannins and polyphenols, *i.e.* geraniin, mallotusinic acid, pedunculagin and agrimoniin, and also EGCG significantly inhibitied mutagenicity of Trp-P-1 and MNNG. These polyphenols also remarkably inhibited *N*-OH-Trp-P-2, a direct-acting mutagen [34].

##### 2.5.2.2. Inhibition of tumor promotion

Among screened polyphenols several tannins, ellagitannins and their oxidized congeners, pentagalloylglucose and EGCG, showed significant inhibition of tumor promotion which is the second stage of two stage chemical carcinogenesis [14,85]. The inhibitory activity leading to cancer prevention was most extensively investigated on EGCG, revealing its positive effects in this area [12].

##### 2.5.2.3. Host-mediated antitumor activity of ellagitannin oligomers

Tumor growth inhibiting effect achieved by administration either before or after intraperitoneal inoculation of tumor cells, called host-mediated antitumor activity, is exhibited by several ellagitannin oligomers [86]. Among over 100 tannins and related polyphenols screened after discovery of this effect in agrimoniin, the most notable inhibitory effect was found for the macrocyclic dimers oenothein B and woodfordin C. This effect was also found for the macrocyclic trimers oenothein A and woodfordin D, and the macrocyclic tetramer woodfordin F. This effect is attributable to the immune response of host animals, as shown by stimulation of interleukin 1 (IL-1) production from human peripheral macrophages by these ellagitannin oligomers [87].

### 2.6. Caffeic Acid Esters (Caffetannins)

Caffetannins are formed by esterification of quinic acid with several molecules of caffeic acid or by mutual esterification between caffeic acids. They can be classified as type A although their activities as tannins are moderate.

#### 2.6.1. Caffeoyl esters of quinic acid (Figure 10).

*Monocaffeoyl quinic acid.* The name caffetannin was applied to chlorogenic acid (5-caffeoylquinic acid) and its congeners in coffee beans [88]. The protein-binding activity of this compound, however, was found low and the tannin activities of east-Asian medicinal plants of *Artemisia* species were found to be mainly attributable to that of 3,5-di*-O*-caffeoylquinic acid and its isomers [89].

**Figure 10:**
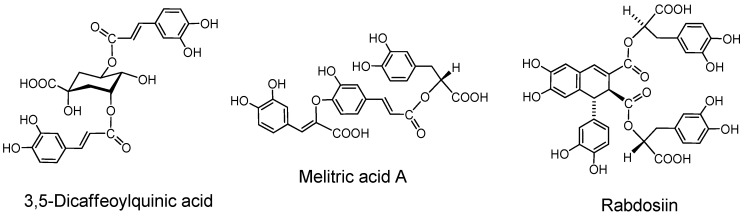
Caffetannin and caffeic acid oligomers.

*Oligocaffeoylquinic acids.* 3,5-Di-*O*-caffeoylquinic acid, its isomers and three isomers of tri-*O*-caffeoylquinic acids [90] can also be counted among the type A tannins.

#### 2.6.2. Caffeic acid oligomers

Rosmarinic acid, a caffeic acid dimer widely distributed in labiate plants and therefore alled labiataetannin, has negligible tannin activities [90]. Lithospermic acid [91], melitric acids A and B [92] are trimers, and lithospermic acid B [92], rabdosiin (Figure 10) and its isomer [93] are tetramers of caffeic acid.

### 2.7. Stilbenoids (Figure 11).

Stilbenoids have been found to form oligomers up to hexamers, often in the plants grown in southern Asia and nearby areas, particularly those used in traditional medicines [94,95,96,97]. They are found in bryophytes and pteridophytes, besides gymnosperms and angiosperms [98]. Stilbenoids are similar to the type A tannins in their recently found properties and activities such as antibacterial activity against methicillin-resistant *Staphyllococcus aureus* (MRSA), antitumor effects and induction of apoptosis, and also in the structural correlations among their oligomers. Various stilbenoid oligomers are known and can be analyzed by HPLC [99].

#### 2.7.1. Stilbenoid monomers and their glucosides

Some stilbenoids such as resveratrol, pterostilbene, and piceatannol are attracting interest because of their antitumorigenic and antileukemic activity, and their occurrence in grape, wine and peanut, although their concentration in wine is low [100]. These stilbenoids are also found in *Vaccinium* berries [101]. The presence of their glucosides in plants has also been found besides the long-known stilbenoid glucosides in *Vaccinium* berries and *Rheum* rhizomes [102].

*Resveratrol (3,4’,5-trihydroxy-trans-stilbene).* This compound has a structural and bioactivity correlation with piceatannol, which is called a tannin [5]. Resveratrol is found in the skin of red grapes, and is a constituent of red wine. It was first isolated from a medicinal plant, *Veratrum album* var. *grandiflorum* [18], and is produced, along with its glucoside and hydroxyveratrol, in *V. album* and polygonaceous plants (*Polygonum cuspidatum,* *P. multiflorum, Reynoutria japonica* and *R. sachalinensis*, *etc.*). It is known as one of the antioxidant polyphenols. Resveratrol has been found to inhibit accumulation of peroxidized lipids in the liver of hepatopathy patients, and lipid peroxidation induced by ADP and NADPH in rat liver microsomes [103], the same system as that applied to hydrolyzable and condensed tannins [10]. Resveratrol has been known as a phytoalexin produced naturally when plants are under attack by pathogens such as bacteria and fungi.

*Piceatannol (3,3’,4,5’-tetrahydroxy-trans-stilbene).* The name piceatannol was originally given to a stilbenoidal aglycone obtained from a glucoside isolated from the bark of a spruce tree (*Picea* species) and regarded as representing the tannin in this bark [5]. The structure now recognized for piceatannol is 3,3’,4,5’-tetrahydroxy-*trans*-stilbene [98,104]. Piceatannol isolated from the seeds of *Euphorbia* *lagascae* is an active 9PS and 3PS (P-388) murine antileukemic agent [19], and has antileishmanial activity [105]. Various activities related to antioxidative action such as inhibition of melangenesis [106] were reported. Apoptosis [107] and other activities were also found. The presence of piceatannol in grape and wine, particularly in red wine as a metabolite of resveratrol, has caused research interest in piceatannol as an anti-cancer and anti-EBV drug. The LMP2A, a viral protein-tyrosine kinase implicated in leukemia, non-Hodgkin’s lymphoma and other diseases associated with Epstein-Barr virus, was blocked *in vitro* by piceatannol [108,109].

*4’-Methoxy-3,3’,5-trans-stilbenetriol 3-O-Glucoside (Rhaponticin) and 3,5,4’-trihydroxystilbene-4’-glucoside.* These stilbenoid glucosides in found in rhubarbs (rhizomes of *Rheum* species, particularly *R. rhaponticum* L. and *R. undulatum* L.), east-Asian medicinal plants, facilitated the discrimination of these species of rhubarb on TLC by their fluorescence under ultraviolet lamp irradiation. Rhaponticin was reported to improve glucose and lipid metabolism [110].

*Flavonostilbenes.* A flavonostilbene with antibacterial activity against methicillin-resistant *Staphylococcus aureus* was isolated from *Sophora leachiana* [111]. Flavonostilbenes, jezonocinols A, B and C with DPPH radical scavenging activity, were isolated from *Picea jezoensis* var. *jezoensis* [112].

**Figure 11:**
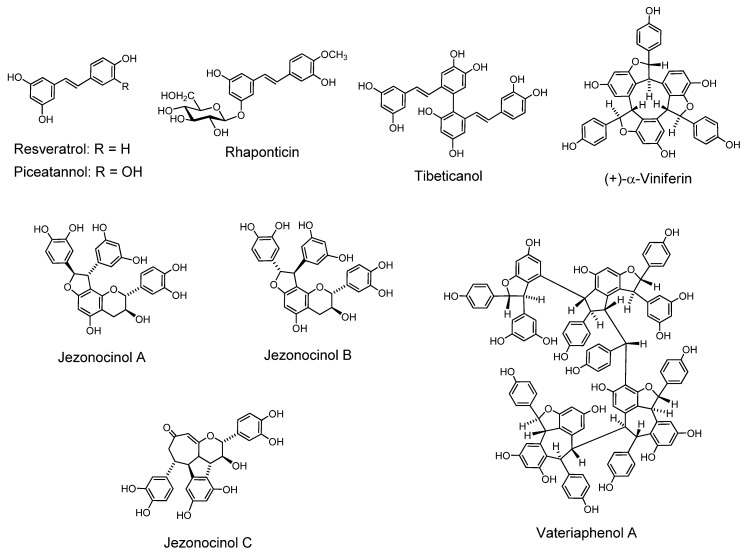
Stilbenoids.

#### 2.7.2. Stilbenoid oligomers

In recent years stilbenoid oligomers of various structures with different extents of oligomerization have been isolated from plants of the Dipterocarpaceae, Gnetaceae, Vitaceae, Cyperaceae, Welwitsiaceae and Leguminosae grown in southern Asia and nearby areas [113].

*Tibeticanol.* This piceatannol dimer was isolated from *Caragana tibetica*, which has been medicinally used in western part of China, exhibits superoxide anion scavenging activity [114]. *Viniferins*, which are resveratrol dimers with antioxidant properties [115] and trimers with anti-inflammatory activity, were isolated from *Caragana chamlagu* [116]. *Vatalbinosides A–E*. These five resveratrol tetramers, along with 13 known compounds, were isolated from stem of *Vatica albiramis* [117]. 

*Resveratrol Hexamers*. Vaticanol D, a superoxide scavenging hexamer, was isolated from *Vatica rassak* [118]. Resveratrol hexamers were also isolated from *Upuna borneensis* [119] and *Dipterocarpus grandiflorus* [120].

*Resveratrol Octamer*. An octamer vateriaphenol A was isolated from *Vateria indica* [121].

*Biological and Pharmacological Activities of the Oligomers*. Antibacterial activity against vancomycin-resistant *Enterococci* (VRE) and methicillin-resistant *Staphylococcus aureus* (MRSA), and the synergism with antibiotics of the stilbene oligomers [122] and also DNA topoisomerase II inhibitory activity [123] of the oligomers were reported. Resveratrol oligomers showed antitumor effects against human cancer lines [124]. Stilbenoids were also found to inhibit growth of leukemia HL60 cells through induction of apoptosis [125]. Anti-hyperlipidemic activity of the oligostilbenoids isolated from a Thai medicinal plant was reported [126].

### 2.8. Phlorotannins (Figure 12)

The phloroglucinol oligomers, named phlorotannins, having tannin activities are produced in brown algae. In the early stages of phlorotannin research they were isolated only after acetylation or methylation of free phenolic hydroxyl groups in their molecules [127]. 

**Figure 12:**
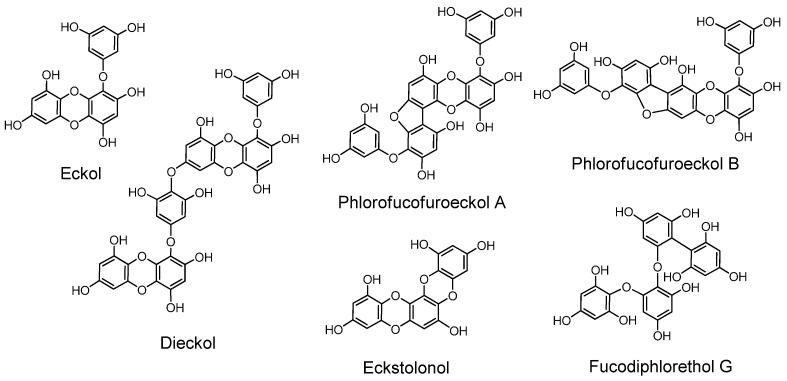
Phlorotannins.

Later, free phlorotannins, exemplified by eckol, an anti-plasmin inhibitor, and phlorofucofuroeckol A, of similar activity, were isolated from *Ecklonia kurome*, an edible brown alga [6,128,129]. Plasma α-2”-macroglobulin was inhibited by the phlorotannins isolated from *E. kurome* [129]. Eckstolonol (5,8,13,14-tetraoxapentaphene-1,3,6,9,11-pentaol) with antioxidant activity [130], along with eckol, phlorofucofuroeckol A and dieckol, were isolated from *E. stolonifera*. Phlorofucofuroeckol-B with anti-allergic activity was isolated from the edible brown alga *Eisenia arborea*, which is occasionally used in Japan as a folk medicine for gynecopathy [131]. Fucodiphlorethol G and dieckol were isolated from *E. cava* [132]. Protection of radiation-induced intestinal injury in mice [133], and other activities of phlorotannin components and eckol were also reported. The biological activities of phlorotannins were masked by acetylation or methylation [134]. The distribution of phlorotannins in *Ecklonia* and *Eisenia* species in Japanese laminariaceous algae has been reviewed [135], and high-molecular-weight phlorotannins from brown algae were reported [136]. Ecological interactions of phlorotannins and other constituents of brown algae were also reviewed [137].

## 3. Type B Tannins: Variable Mixtures of Analogous Polyphenols

The structures and compositions of this type of tannins obtainable from a particular species of plant are not always the same, as their structures and compositions vary seasonally, and also depending on the growth conditions of the plants, and on the methods used to produce the preparations.

### 3.1. Gallotannins of Type B

The Type B gallotannins often have additional galloyl groups bound with depsidic linkages to each galloyl group on the carbohydrate or quinic acid nucleus. Their examples are Chinese gallotannin and Turkish gallotannin [138]. They belong to the hydrolyzable tannins group.

The tannin preparations registered under the name tannic acid in the pharmacopoeias of many countries, and those appearing in the textbooks of medical education without showing chemical characterization are mostly type-B gallotannins.

### 3.2. Polyhydroxyflavan Oligomers (Condensed Tannins)

More complex mixtures are the condensed tannins which are galloylated or non-galloylated polyhydroxyflavan oligomers. Most plant polyhydroxyflavans having tannin-like activities except EGCG and ECG are condensed tannins which generally exhibit the activities by complex oligomerization, although the highly condensed ones, called phlobaphene, are insoluble and therefore inactive. Black tea tannins are also mixtures of variable composition produced by fermentation of tea leaves, although some lower molecular condensates such as theaflavin and its derivatives can be isolated [139]. Some polyhydroxyflavans with lower degrees of condensation found in plants, e.g., some procyanidins and prodelphinidins, are isolable, but they change their mode and extent of condensation seasonally, and the bio-activities of these labile oligomers in condensed tannins are often evaluated as the mixtures. Condensed tannins in fruits generally increase their extent of condensation upon ripening, as found in kaki fruits [140]. Chemical approaches to the small condensates in these often heterogeneous oligomers [141] are contributing to the elucidation of the complex features of polyhydroxyflavan oligomers [142].

#### 3.2.1. Galloylated polyhydroxyflavan oligomers

Occurrence of condensed tannins of this type is limited to the plants of Dicotyledoneae where gallic acid can be biosynthesized. The biological activities of these tannins are generally significantly higher than those of non-galloylated polyhydroxyflavans [2,143]. Most of the condensed tannins contained in traditional medicinal plants as their main components are galloylated at *O*-3 to a variable extent.

Highly Galloylated Polyhydoxyflavan Oligomers in *Saxifraga stolonifera*. The herb *Saxifraga stolonifera* is a folk medicine used in Japan for treating earache, painful hemorrhoids, wounds and swelling. The condensed tannin fraction named Ss-tannin 1 extracted from this plant was the most highly galloylated (96%) among the galloylated polyhydroxyflavans found in medicinal plants [144].

Galloylated Polyhydroxyflavan Oligomers in *Diospyros kaki*. The fruit of *Diospyros kaki,* rich in partially (70–80%) galloylated condensed tannins [140], changes to be a sweet autumn fruit by the progress of condensation of its tannin yielding insoluble high molecular polymers. However, the highly astringent juice of unripe green fruits, after fermentation, has been used as a folk medicine in Japan.

#### 3.2.2. Non-galloylated polyhydroxyflavans

Non-galloylated polyhydroxyflavans in terrestrial plants are mostly produced in Gymnosperms and Monocotyledons 

## 4. Conclusions

Extensive chemical, biological and pharmacological investigations of tannins, particularly those of the ellagitannins and their congeners, and also of related polyphenols of various constant structures, are changing the concept and significance of tannins in human life. These discoveries are opening new ways to classify and study the chemical, biological and pharmacological properties of tannins.
